# Dual-layer dual-energy CT characterization of thrombus composition in acute pulmonary embolism and chronic thromboembolic pulmonary hypertension

**DOI:** 10.1007/s10554-024-03309-2

**Published:** 2024-12-25

**Authors:** Roman Johannes Gertz, Simon Lennartz, Kenan Kaya, Robert Peter Wawer Matos Reimer, Lenhard Pennig, Jonathan Kottlors, Jan Robert Kröger, Carsten Herbert Gietzen, Nils Große Hokamp, Stephan Rosenkranz, Florian Johannes Fintelmann, Michael Pienn, Alexander Christian Bunck

**Affiliations:** 1 Department of Radiology, Faculty of Medicine and University Hospital Cologne, University of Cologne, Kerpener Str. 62, 50937 Cologne, Germany; 2 Department of Radiology, Neuroradiology and Nuclear Medicine, Johannes Wesling University Hospital Minden, Ruhr University Bochum, Bochum, Germany; 3 Department of Cardiology, Heart Center, Faculty of Medicine, University of Cologne, Cologne, Germany; 4 Thoracic Imaging & Intervention, Massachusetts General Hospital, Boston, USA; 5 Ludwig Boltzmann Institute for Lung Vascular Research and Division of Pulmonology, Medical University of Graz, Graz, Austria

**Keywords:** Dual-Layer Dual-Energy computed tomography, Material decomposition, Acute pulmonary embolism, Chronic thromboembolic pulmonary hypertension

## Abstract

To evaluate dual-layer dual-energy computed tomography (dlDECT)-based characterization of thrombus composition for differentiation of acute pulmonary embolism (PE) and chronic thromboembolic pulmonary hypertension (CTEPH). This retrospective single center cohort study included 49 patients with acute PE and 33 patients with CTEPH who underwent CT pulmonary angiography on a dlDECT from 06/2016 to 06/2022. Conventional images), material specific images (virtual non-contrast [VNC], iodine density overlay [IDO], electron density [ED]), and virtual monoenergetic images (VMI_50KeV_) were analyzed. Regions-of-interest (ROIs) were manually placed in pulmonary artery thrombi, and morphological imaging characteristics for acute and chronic PE were assessed. Area under the receiver operating characteristics curve (AUC) of ROI measurements, morphological imaging features, and their combination in distinguishing between acute PE and CTEPH were evaluated. Compared to PE, thrombi in patients with CTEPH had lower attenuation on conventional images (Median [inter-quartile range]: 40 [35–47] HU vs 64 [52–83] HU) and VMI_50keV_ reconstructions (59 [46–72] HU vs 101 [80–123] HU) as well as decreased iodine uptake (IDO: 0.5 [0.2–1.0] vs 1.2 [0.5–1.8]; p for all < 0.001). Conventional images and VMI_50keV_ reconstructions were the most accurate for differentiating between acute and chronic thrombi (conventional: AUC 0.92, 95% CI 0.86–0.98; VMI_50keV_: AUC 0.91, 95% CI 0.85–0.97). Main pulmonary artery (MPA) diameter combined with thrombus attenuation significantly increased the AUC compared to MPA diameter alone (*p* = 0.002 respectively). Thrombi in patients with CTEPH exhibit lower attenuation and reduced contrast enhancement. Analyzing attenuation in pulmonary thrombi may add diagnostic information to established morphological parameters in differentiating acute PE from CTEPH.

**Supplementary Information:**

The online version contains supplementary material available at 10.1007/s10554-024-03309-2.

## Introduction

Acute pulmonary embolism (PE) is the third most common cause of cardiovascular death after coronary artery disease and stroke [1–3]. A less common, yet grave complication of unresolved acute PE or recurrent subclinical pulmonary emboli is chronic thromboembolic pulmonary hypertension (CTEPH) [4–7]. The actual incidence of CTEPH remains a subject of debate, varying from roughly 0.6% in “all-comers” populations to about 3% among those who have survived PE [7].

It is widely recognized that thrombi undergo structural changes over time, stemming from the intricate interactions among coagulation factors, cytokines, leukocytes, and various other elements. While acute thrombi are predominantly composed of a loose mesh of fibrin and red blood cells (RBCs), chronic clots develop a fibrotic collagenous framework, ultimately becoming primarily acellular connective tissue [8]. This ongoing organization affects the outer appearance of the thrombus which is why the CT-based differentiation between acute and chronic pulmonary emboli routinely relies on morphological features [9]. While acute thrombi present as central filling defects (“polo-mint” or “railway” sign) or eccentric filling defects with an acute angle to the vessel wall, chronic thrombus features encompass thrombus lamination with obtuse angles to the contrast column, abrupt vessel narrowing with intimal irregularities, ‘webs and bands’, and post-stenotic dilatation [9–14]. However, these findings are challenging to recognize, particularly for radiologists with limited experience in PH imaging [15], as evidenced by low inter-reader agreement, including among experts [16]. Additionally, the specificity of these diagnostic parameters is limited. Notably, 15% of patients with acute PE show signs of chronicity or CTEPH at baseline imaging [16]. Nonetheless, a rapid and accurate diagnosis is essential, as it guides treatment decisions and untreated CTEPH patients are at higher risk of death [17, 18].

By acquiring two spectrally distinct datasets, dual-energy CT (DECT) enables the computation of material specific maps such as virtual non-contrast images (VNC) and iodine density overlay (IDO) maps, which both rely on material decomposition [19]. Furthermore, DECT allows for the reconstruction of energy-specific images (virtual monoenergetic images (VMI)), approximating images from an acquisition with a monoenergetic X-ray beam [20–22].

There is evidence that clot attenuation on unenhanced CT scans is not only strongly associated with histology [23, 24] but also with therapeutic outcome in stroke and deep vein thrombosis [25, 26]. In vitro studies suggest that DECT-based spectral reconstructions can further increase the diagnostic abilities of CT to differentiate between acute and chronic thrombi [27]. However, factors such as variances in contrast-material timing, vessel anatomy, intravascular pressure, thrombus origin (arterial vs. venous), thrombus size and configuration, bring into question the applicability of stroke imaging findings to large vessel occlusions in the venous and the pulmonary arteries [26, 28]. This uncertainty is mirrored in the indeterminate outcomes of existing studies [29–32], which either contrast with findings from in vitro and histopathological research or differ from results in stroke imaging, where (DE)CT-derived thrombus composition has been studied much more extensively [23].

The objective of this study was to quantitatively assess dual-layer DECT (dlDECT)-derived thrombus characteristics in both acute PE and CTEPH. We sought to determine their diagnostic capability in identifying and distinguishing patients with acute PE and CTEPH.

## Materials and methods

### Study population

This study was approved by the local institutional review board (Ethics Committee of the Faculty of Medicine from the University of Cologne, Cologne, Germany). The necessity for informed consent was waived due to the retrospective design of the study. All clinical investigations were conducted in accordance with the Declaration of Helsinki.

This was a single-center, retrospective study. All patients screened for study eligibility were consecutive patients who underwent CTPA on the same dlDECT (IQon, Philips Healthcare) between June 2016 and June 2022 as part of their routine clinical work-up for suspected acute PE or CTEPH. These patients were identified through a structured search within the radiological information system and the picture archiving and communications system.

The final diagnosis of CTEPH was established by expert consensus based on all available diagnostic tests, including right heart catheterization, ventilation/perfusion scintigraphy and CT imaging in accordance with the 2015 ESC/ERS guidelines [33]. Patients with suspected CTEPH had to be anticoagulated for three months prior to referral. Inclusion criteria for patients with acute PE were: (1) suspicion of acute PE based on patient´s medical history and (2) concordant imaging findings on CTPA.

Exclusion criteria for both patient groups encompassed artifacts affecting the thrombus and non-thrombotic occlusion (e.g., tumorous occlusion) on CTPA. Specific to the acute PE group, exclusion criteria were: (1) a history of acute PE, known chronic thromboembolic disease, or vascular signs of chronicity (laminated thrombus with obtuse angle to the contrast column or calcification, intravascular webs, complete arterial occlusion, arterial narrowing or retraction, post-stenotic vascular dilatation [9]), (2) thrombus size ≤ 12.5 mm^2^. For the CTEPH group, exclusion criteria included the absence of detectable thrombus (i.e., those presenting only other signs of chronicity) or a thrombus size ≤ 12.5 mm^2^.

### Image acquisition and reconstruction

All patients received an intravenous 50 ml bolus of contrast media (300 mg iodine/ml, Accupaque, GE Healthcare) followed by a 40 ml NaCl chaser, with a flow rate of 4 ml/s. After reaching an attenuation of 150 HU in the main pulmonary artery (MPA), scanning was initiated with a delay of 4.9 s in craniocaudal direction. The acquisition parameters were as follows: slice collimation 64 × 0.625 mm; rotation time 0.33 s; tube potential 120 kV; tube current 75 mAs_ref_ with activated automatic tube current modulation. For all reconstructions, a dedicated spectral reconstruction algorithm with a soft tissue kernel was used (Spectral, B, Philips Healthcare). Images were reconstructed in axial orientation every 0.5 mm with a slice thickness of 1 mm. Matrix was set to 512 × 512.

### Postprocessing

The same spectral dataset, with a section thickness of 2 mm and a section increment of 1 mm, was employed to reconstruct conventional images, IDO maps, VNC, Z-effective, VMI_50KeV_ and electron-density images using a dedicated image viewer (IntelliSpace Portal, version 9.0; Philips). Conventional images were derived using a hybrid iterative reconstruction algorithm (iDose 4, level 3; Philips). The VMI_50keV_ were acquired using a proprietary spectral reconstruction algorithm (Spectral B, level 3; Philips). The IDO maps were reconstructed as quantitative color-coded iodine maps.

### Image analysis

#### Morphological CTPA analysis

##### Assessment of thrombus morphology

**Fig. 1:**
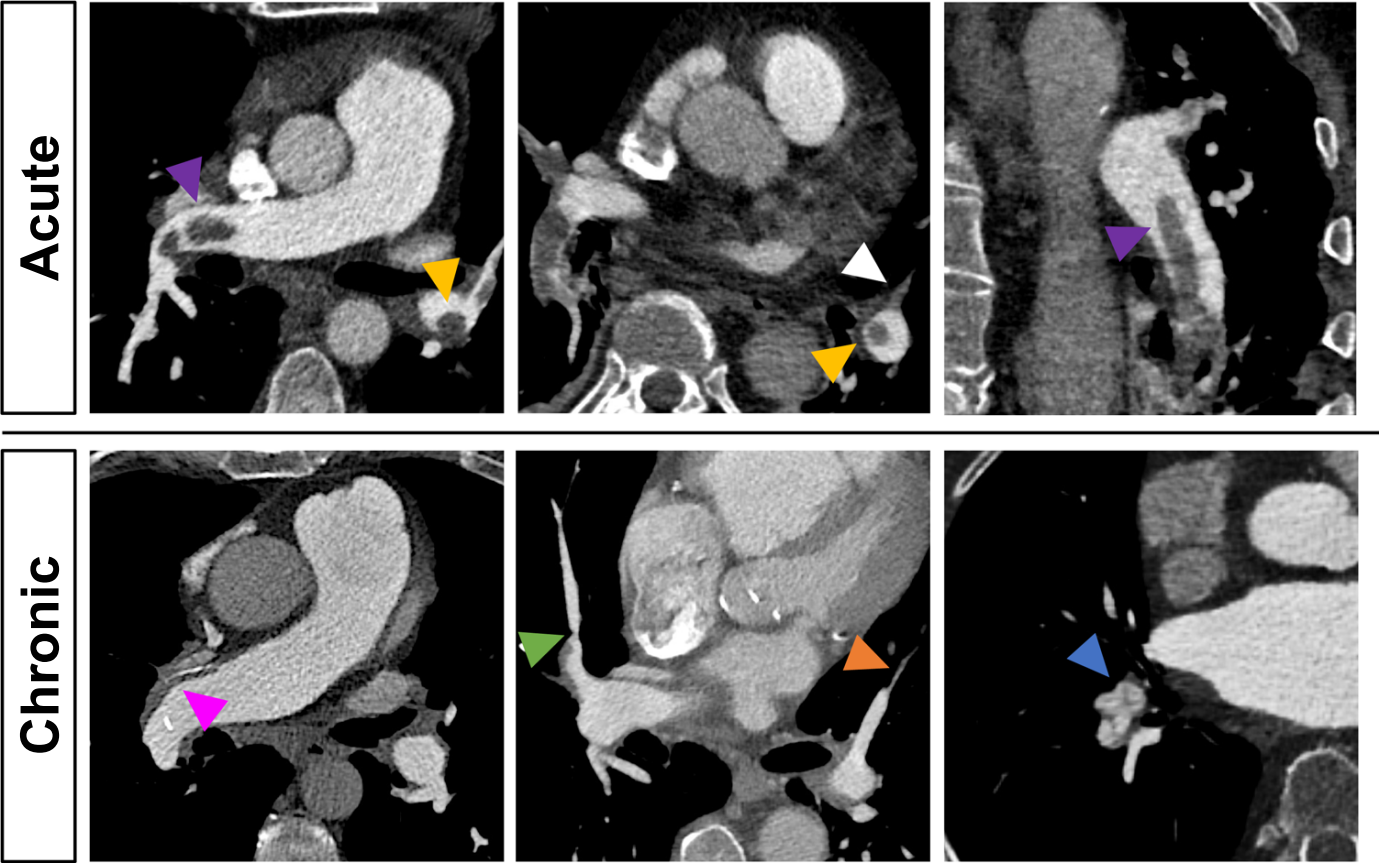
Comparison of thromboembolic imaging features between acute and chronic PE. Acute PE: Central or eccentric filling defects surrounded by high-attenuation contrast medium (“polo-mint sign”—yellow arrowheads and “railway sign”—purple arrowheads) and complete arterial occlusion without lumen retraction (white arrowhead). Chronic PE: Laminated thrombus with calcifications (pink arrowhead), post-stenotic dilatation (green arrowhead), vascular retraction (orange arrowhead), and intravascular webs (blue arrowhead)

A radiologist with 4 years of experience in cardiovascular imaging (R.J.G.) assessed imaging findings indicating PE and CTEPH in accordance with the prevailing evidence available at the time the study was designed [9–11, 17]. These included: thrombus morphology/direct vascular features (e.g., “polo-mint” or “railway” sign, laminated thrombus), indirect vascular features (e.g., MPA dilatation, dilatated bronchial arteries), indirect cardiac features (e.g., right-ventricular (RV) dilatation or hypertrophy) and indirect parenchymal features (e.g., pulmonary infarction). Figure 1 illustrates typical examples of acute and chronic thromboembolic imaging features. All morphological imaging features are listed in Table 1.

**Table 1:** Comparison of morphological imaging features in acute PE and CTEPH

	Acute PE	CTEPH	*p*
*Direct vascular features*			
Acute Angle (no/yes; *n* = 80; *n* = 47/33)	35/12	32/1	0.02
Polo-Mint/Railway Sign (no/yes, *n* = 80; *n* = 47/33)	16/31	31/2	< 0.001
Lumen Distension (no/yes, *n* = 80; *n* = 47/33)	34/13	33/0	0.003
Laminated Thrombus (no/yes, *n* = 80; *n* = 47/33)	47/0	5/28	< 0.001
Thrombus Calcification (no/yes, *n* = 80; *n* = 47/33)	47/0	25/8	0.0015
Reperfusion Channel (no/yes, *n* = 80; *n* = 47/33)	47/0	28/5	0.02
Intravascular Webs (no/yes, *n* = 80; *n* = 47/33)	47/0	10/23	< 0.001
Vascular Retraction (no/yes, *n* = 80; *n* = 47/33)	47/0	21/12	< 0.001
Poststenotic Vascular Dilatation (no/yes, *n* = 80; *n* = 47/33)	47/0	23/10	< 0.001
*Indirect vascular features*			
Diameter MPA (mm; *n* = 80; *n* = 47/33)	30 [27–33]	37 [34–40]	< 0.001
Diameter Ao (mm; *n* = 80; *n* = 47/33)	33 [30–37]	34 [31–37]	0.7
Diameter ratio MPA/Ao (1; *n* = 80; *n* = 47/33)	0.88 [0.81–0.96]	1.04 [1.00–1.19]	< 0.001
RV Diameter 4CV^a^ (mm; *n* = 80; *n* = 47/33)	47 [42–54]	56 [48–62]	0.002
LV Diameter 4CV^a^ (mm; *n* = 80; *n* = 47/33)	41 [35–50]	45 [36–49]	0.7
Diameter Ratio RV/LV (1; *n* = 80; *n* = 47/33)	1.16 [0.91–1.47]	1.31 [1.02–1.56]	0.13
IVS Flattening^a^ (no/yes)	29/18	11/22	0.02
RV Hypertrophy^b^ (no/yes)	47/0	15/18	< 0.001
Diameter Bronchial Arteries (mm; *n* = 60; *n* = 30/30)	1.6 [1.4–1.8]	2.6 [2.3–3.1]	< 0.001
Pathologic or Dilatated Bronchial Arteries^c^ (no/yes/lc/nd)	25/5/3/14	1/29/3/0	< 0.001
**Parechymal features**			
Mosaic Perfusion (no/yes, *n* = 80; *n* = 47/33)	47/0	19/14	< 0.001
Parenchymal Bands (no/yes, *n* = 80; *n* = 47/33)	47/0	21/12	< 0.001
Pulmonary Infarction (no/yes; *n* = 80; *n* = 47/33)	35/12	32/1	0.02
Thrombus Level (cent./segm./sub-segm.)	28/19/0	28/5/0	0.03

Categorial data are given as n/n. Continuous data are given as median [IQR]

^a^on a multiplanar-reformatted four-chamber view

^b^characterized by free wall-thickness > 4 mm

^c^defined as > 1.5 mm

*PE* pulmonary embolism, *CTEPH* chronic thromboembolic pulmonary hypertension, *MPA* main pulmonary artery, *Ao* Aorta, *RV* right ventricle/ventricular, *LV* left ventricle/ventricular, *IVS* interventricular septum, *lc* too low aortic contrast to identify bronchial arteries, *nd* not detectable/too small for measurement

##### Assessment of thrombus level

Thrombus level was assessed and classified based on the largest arteries involved, as previously described [34, 35]. Utilizing Boyden´s nomenclature [36] thrombi were classified as either central (lobar or main pulmonary arteries, with or without segmental or subsegmental arteries), segmental (with or without subsegmental arteries), or subsegmental.

#### Quantitative analysis

A radiologist with 4 years of experience in cardiovascular imaging (R.J.G.) identified two axial slices depicting the largest extent of the thrombus, ensuring these were unaffected by artifacts, e.g., beam-hardening due to contrast in the subclavian vein. The radiologist placed a circular region-of-interest (ROI) covering the largest possible area of the thrombus. To ensure representative capturing of attenuation characteristics, each ROI exceeded 12.5 mm^2^ with enough distance from adjacent structures to avoid partial volume effects. Patients with thrombi that did not allow for sufficiently large ROIs were excluded from the study. In case of thrombus calcification, ROIs were drawn outside the calcified areas.

ROIs were placed in conventional images and then copied to IDO, VNC, Z-effective, VMI_50KeV_ and electron-density images to ensure equal localization and size. Mean values for attenuation and the respective spectral reconstructions were obtained. All measurements were performed on a dedicated workstation (IntelliSpace Portal; Philips).

### Reproducibility

To assess intra-observer reproducibility the same observer repeated the analysis four weeks-later in a randomly chosen cohort of 10 patients with acute PE and 10 CTEPH patients. Inter-observer reproducibility was derived from the analysis of a second skilled observer (K.K.) with three years of experience in cardiovascular imaging. Both readers were blinded to the clinical data of the patients as well as to the results of the other observer.

### Statistical analysis

Statistical analysis was performed in R (R Core Development Team, version 4.3.2), using RStudio (RStudio, Version 2023.06.1). Differences in continuous readouts between groups were analyzed using the Mann–Whitney U test. Differences in categorical data were assessed with Pearsons Χ^2^ test. The diagnostic accuracy was assessed by determining the area under the receiver operating characteristic curve (AUC) using the pROC package [37]. Optimal sensitivity and specificity thresholds were identified using Youden’s index. To evaluate if quantitative readouts derived from the thrombus composition offer additional information for distinguishing acute PE from CTEPH, we combined them with diameters of bronchial arteries and the MPA, as well as the ratio of MPA to ascending aorta diameters, following the methodology proposed by Pepe et al. [38]. Differences in AUCs were assessed using the DeLong test [39]. Intra- and inter-reader consistency and agreement were analyzed with inter-class correlation coefficients (ICC) [40, 41] using the psych package [42] Bland–Altman analysis [43]. Results are presented as median [inter-quartile range]; p-values < 0.05 were considered statistically significant.

## Results

### Patient demographics

**Fig. 2:**
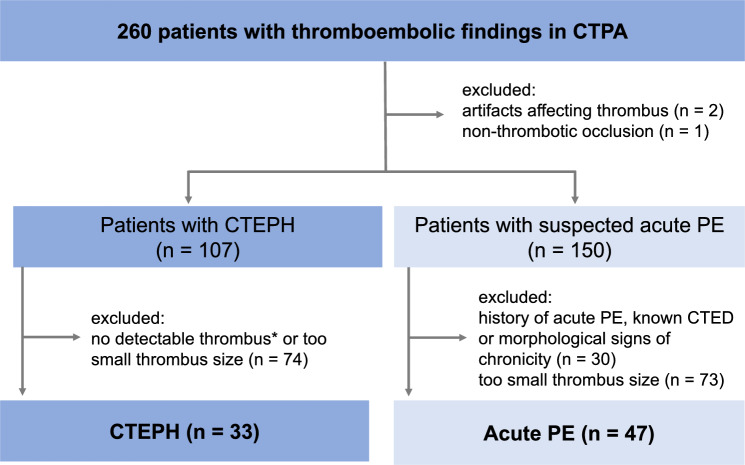
Study flow chart. *CTPA* CT pulmonary angiography, *CTEPH* chronic thromboembolic pulmonary hypertension, *PE* pulmonary embolism, *CTED* chronic thromboembolic disease. *Only chronic thromboembolic findings other than chronic thrombi (e.g., vascular retraction)

Out of 260 patients with thromboembolic findings 3 were excluded due artifacts affecting the thrombus (*n* = 2) or tumorous vascular occlusion (*n* = 1). Further, from the CTEPH group (*n* = 107) 74 patients were excluded because of no detectable thrombus or too small thrombus size. Similarly, from the acute PE group (*n* = 150), 30 patients were excluded due to patient history of prior PE or morphological signs of chronicity and 73 patients for having a too small thrombus size. Thus, the final dataset comprised of 33 patients with CTEPH and 47 patients with acute PE (Fig. 2). There were no differences between the groups regarding age (median [inter-quartile range]: CTEPH, 62 [49–76] years vs acute PE, 60 [49–72] years, *p* = 0.4) or sex (CTEPH, 19/14 m/f vs acute PE, 23/24 m/f, *p* = 0.6). Mean attenuation of the MPA was similar in both groups (CTEPH, 329.0 HU [281.0–417.0] vs acute PE, 352.0 [286.0–413.0], *p* = 0.79).

### Morphological imaging features

Morphological imaging features are detailed in Table 1. Three patients with CTEPH displayed morphological features characteristic of acute PE. However, these features were significantly more prevalent in the acute PE patient group (*p* < 0.05 for all comparisons).

Patients with CTEPH exhibited a larger MPA diameter compared to those with acute PE (37.0 [34–40] mm vs 30 [27–33] mm; *p* < 0.001). Additionally, the MPA/Aorta ratio was higher in CTEPH patients (1.04 [1.00–1.19] vs 0.88 [0.81–0.96], *p* < 0.001). Observations of intraventricular septum flattening and RV hypertrophy were more frequent in the CTEPH group (*p* = 0.02 and *p* = 0.001, respectively). Furthermore, CTEPH patients had larger bronchial artery diameters (2.6 [2.3–3.1] mm vs 1.6 [1.4–1.8] mm, *p* < 0.001). Among the CTEPH cohort, 12 patients showed signs of resolved pulmonary infarction, while 12 patients in the acute PE group exhibited signs of acute pulmonary infarction. Regarding the location of thrombi, segmental thrombi were more prevalent in the acute PE group (*p* = 0.03).

### dlDECT-derived thrombus characterization

**Fig. 3:**
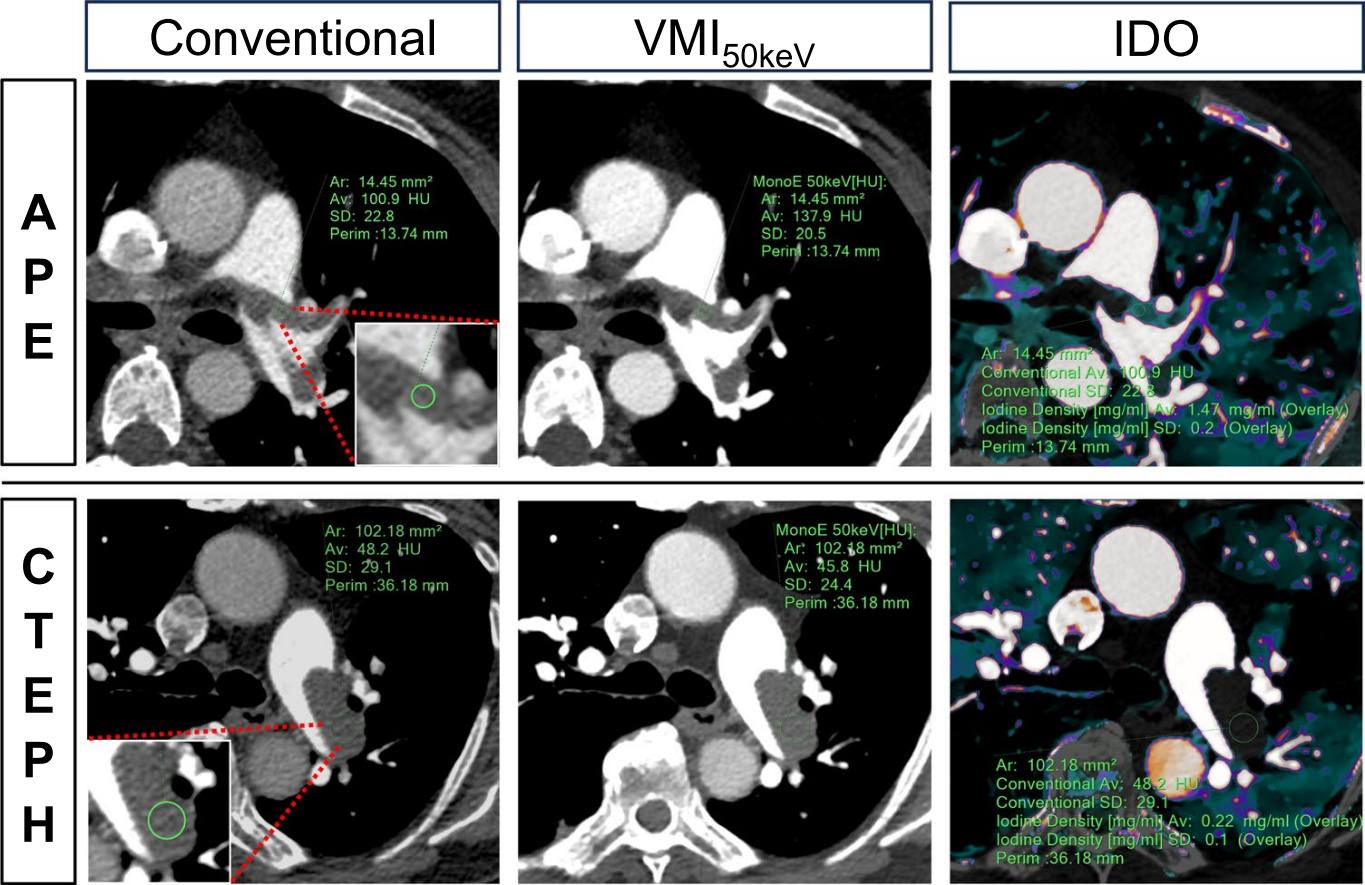
Comparative quantitative analysis of thrombus composition using dlDECT in a patient with acute PE and a patient with CTEPH, both exhibiting central thrombi. *APE* acute pulmonary embolism, *CTEPH* chronic thromboembolic pulmonary hypertension, *VMI* virtual monoenergetic images, *IDO* iodine density overlay

Figure 3 presents a comparative quantitative analysis, utilizing dlDECT to assess thrombus material decomposition, in a patient with acute PE and a patient with CTEPH, both exhibiting central thrombi.

Thrombi in patients with CTEPH revealed a significantly lower attenuation than those in acute PE, both in conventional images (40 [35–47] HU vs 64 [52–83] HU, *p* < 0.001) and VMI_50keV_ reconstructions (59 [46–72] HU vs 101 [80–123] HU). Further, chronic thrombi showed decreased iodine uptake in IDO reconstructions (0.5 [0.2–1.0], vs 1.2 [0.5–1.8], *p* < 0.001) and a lower effective atomic number (7.5 [7.4–7.8] vs 8.0 [7.6–8.4], *p* < 0.001). VNC attenuation and electron-density did not differ between groups (*p* = 0.09 and 0.08 respectively), as detailed in Table 2.

**Table 2:** Comparison of dlDECT-derived quantitative thrombus analysis in acute PE vs CTEPH

	Acute PE	CTEPH	*p*
Conventional (HU; *n* = 47/33)	64 [52–83]	40 [35–47]	< 0.001
VMI_50KeV_ (HU; *n* = 47/33)	101 [80–123]	59 [46–72]	< 0.001
IDO (mg/ml; *n* = 47/33)	1.2 [0.5–1.8]	0.5 [0.2–1.0]	< 0.001
VNC (HU; *n* = 47/33)	33 [24–50]	29 [22–36]	0.09
Z-Effective (1; *n* = 47/33)	8.0 [7.6–8.4]	7.5 [7.4–7.8]	< 0.001
Electron Density (%, *n* = 33/22)	104 [103–105]	103 [103–104]	0.08
ROI (mm^2^; *n* = 47/33)	28 [20–50]	34 [22–66]	0.2

Data presented as median [inter-quartile range]. n, number of subjects with acute PE/CTEPH, respectively

*PE* pulmonary embolism, *CTEPH* chronic thromboembolic pulmonary hypertension, *HU* Hounsfield units, *VMI* virtual monoenergetic images, *IDO* iodine density overlay, *VNC* virtual non-contrast

**Fig. 4:**
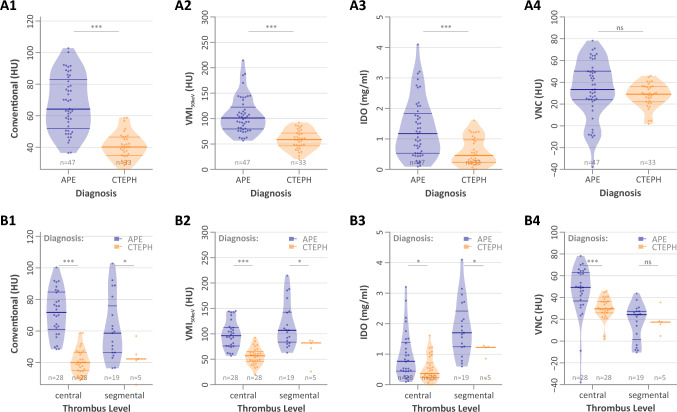
Comparison of thrombus properties between patients with acute pulmonary embolus and chronic thromboembolic pulmonary hypertension. Violin plots display the distribution of thrombus properties in the overall cohort (Panels A1-A4) and according to central vs. segmental thrombus location (Panels B1-B4). Thick lines indicate median values, while thin lines represent lower and upper quartiles. *APE* pulmonary embolism, *CTEPH* chronic thromboembolic pulmonary hypertension, *VMI* virtual monoenergetic images, *IDO* iodine density overlay, *VNC* virtual non-contrast

These results were paralleled by the subanalysis for segmental thrombi. However, when focusing on thrombi with central location only, central thrombi also differed in their attenuation in VNC reconstructions, with central acute thrombi revealing higher attenuation than central chronic thrombi (49 [37–63] vs 30 [26–36], *p* < 0.001) (Table 3, Fig. 4).

**Table 3:** Comparison of dlDECT-derived quantitative thrombus analysis in acute PE vs CTEPH by thrombus level

	Central thrombi		*p*	Segmental Thrombi		*p*
	Acute PE	CTEPH		Acute PE	CTEPH	
Conventional (HU; *n* = 28/28; 19/5)	72 [61–85]	40 [35–47]	*p* < 0.001	59 [46–76]	42 [41–45]	*p* = 0.03
VMI_50KeV_ (HU; *n* = 28/28; 19/5)	96 [76–113]	57 [45–65]	*p* < 0.001	107 [84–143]	82 [72–86]	*p* = 0.03
IDO (mg/ml; *n* = 28/28; 19/5)	0.8 [0.4–1.4]	0.4 [0.2–0.7]	*p* = 0.02	1.7 [1.2–2.4]	1.2 [0.9–1.2]	*p* = 0.04
VNC (HU; *n* = 28/28; 19/5)	49 [37–63]	30 [26–36]	*p* < 0.001	24 [2–27]	17 [15–18]	*p* = 0.8
Z-Effective (1; *n* = 28/28; 19/5)	7.7 [7.5–8.1]	7.5 [7.4–7.6]	*p* = 0.008	8.2 [8.1–8.6]	8.0 [7.8–8.0]	*p* = 0.04
Electron Density (%, *n* = 19/20; 14/2)	105 [104–106]	103 [103–104]	*p* < 0.001	103 [101–103]	102, 102	NA
ROI (mm^2^; *n* = 28/28; 19/5)	48 [30–56]	38 [27–69]	*p* = 0.9	20 [15–27]	17 [16–19]	*p* = 1.0

Data are given as median [IQR]. n, number of subjects with acute PE/CTEPH, respectively, with central thrombi as well as segmental thrombi

*PE* pulmonary embolism, *CTEPH* chronic thromboembolic pulmonary hypertension, *HU* Hounsfield units, *VMI* virtual monoenergetic images, *IDO* iodine density overlay, *VNC* virtual non-contrast

On the basis of AUC analysis conventional and VMI_50keV_ performed best in the differentiation between acute and chronic thrombi (conventional: AUC 92%, 95% CI 86–98%; VMI_50keV_: 91%, 95% CI 85–97%). A threshold of 48 HU in conventional reconstructions provided a sensitivity of 85% and a specificity of 87%. Similarly, a threshold of 73 HU in VMI_50keV_ achieved a sensitivity of 76% and a specificity of 89% (Table 4).

**Table 4:** Diagnostic accuracy of dlDECT-derived quantitative parameters for the differentiation between acute and chronic thrombi

	AUC (95% CI)	Optimal cut-off value (sensitivity/specificity)
Conventional (HU; *n* = 47/33)	92% (86–98%)	48 HU (85%/87%)
VMI_50keV_ (HU; *n* = 47/33)	91% (85–97%)	73 HU (76%/89%)
IDO (mg/ml; *n* = 47/33)	76% (65–86%)	1.3 mg/ml (97%/45%)
VNC (HU; *n* = 47/33)	61% (49–74%)	41 HU (94%/45%)
Z-Effective (1; *n* = 47/33)	77% (67–88%)	8.1 (97%/47%)
Electron Density (%; *n* = 33/22)	64% (50–79%)	105 (95%/42%)

*HU* Hounsfield units, *VMI* virtual monoenergetic images, *IDO* iodine density overlay, *VNC* virtual non-contrast

The diagnostic accuracy for the differentiation of CTEPH and acute PE, as measured by ROC analysis, significantly increased when thrombus attenuation in both conventional and VMI_50keV_ reconstructions was considered. The AUC for evaluating sole the diameter of the MPA was 86% (95% CI 78–94%). This increased to 96% (95% CI 92–100%, *p* = 0.002) when combined with attenuation measurements from conventional images, and similarly to 96% (95% CI 93–100%, *p* = 0.002) when combined with VMI_50keV_ attenuation. Likewise, integrating these parameters with the MPA-to-Aorta diameter ratio resulted in a significant increase in the AUC, as compared to using the diameter ratio alone (*p* = 0.002, respectively, Table 5).

**Table 5:** Comparative analysis of diagnostic accuracy between dlDECT-derived quantitative parameters and morphological CT features

Readout A	Readout B	AUC of ROC analysis		Optimal Combination	*p*	
		Readout A	Readout B		A vs Comb	B vs Comb
Conventional (HU; *n* = 47 vs 33)	Diameter MPA (mm; *n* = 47 vs 33)	92% (86–98%)	86% (78–94%)	96% (92–100%)	0.10	0.002
	Diameter ratio MPA/Ao (1; *n* = 47 vs 33)		83% (74–92%)	96% (92–100%)	0.11	0.002
	Diameter Bronchial Arteries (mm; *n* = 30 vs 30)		95% (89–100%)	99% (98–100%)	0.10	0.2
VMI_50keV_ (HU; *n* = 47 vs 33)	Diameter MPA (mm; *n* = 47 vs 33)	91% (85–97%)	86% (78–94%)	96% (93–100%)	0.03	0.002
	Diameter ratio MPA/Ao (1; *n* = 47 vs 33)		83% (74–92%)	96% (92–100%)	0.04	0.002
	Diameter Bronchial Arteries (mm; *n* = 30 vs 30)		95% (89–100%)	98% (95–100%)	0.03	0.3

*AUC* area under the curve, *ROC* receiver operation characteristic curve, *HU* Hounsfield units, *MPA* main pulmonary artery, *Ao* Aorta, *VMI* virtual monoenergetic images

### Reproducibility

The intra-observer reproducibility found excellent agreement for the individual readouts with one-way random effect model ICCs above 0.9 (Table S1), despite a considerable difference in the ROI sizes applied. Bland–Altman analyses showed constant biases of 2 [− 5–13] HU and 2 [− 18–21] HU for the attenuation in the conventional images and the VMI_50keV_ images, respectively, which is much smaller than the difference between the acute PE and CTEPH groups. Similarly, the inter-observer reproducibility showed a good agreement with ICCs ranging between 0.80 and 0.88 for the individual readouts. Even though for several readouts the one-way model had to be rejected in favor of the two-way random or mixed model, the proximity of these three values suggests that there are no systematic differences between the two readers. Constant bias between reader 1 and 2 was for the conventional image attenuation and the VMI_50keV_ attenuation was 2 [− 11–27] HU and 8 [− 11–44] HU, respectively.

## Discussion

With this study, we aimed to determine the differences in dlDECT-based thrombus properties between patients with acute PE and CTEPH, and to evaluate whether these novel imaging features improve diagnostic accuracy in distinguishing between the two entities. Our findings revealed that chronic thrombi are characterized by lower attenuation in both conventional and VMI_50keV_ reconstructions, along with reduced iodine uptake. Additionally, we found that central chronic thrombi exhibit lower attenuation in VNC images. Importantly, conventional and VMI_50keV_ reconstructions provide significant diagnostic capability in distinguishing between acute PE and CTEPH, further enhancing the diagnostic accuracy of established morphological imaging features.

A broad variety of morphological imaging features has been suggested to differentiate between acute and chronic stages of pulmonary emboli [9–14]. While differentiation between both stages is utterly relevant from a clinical perspective as it defines therapy [18], established radiological parameters fall short in meeting clinical demands for several reasons. First, they lack specificity since a great proportion (as high as 89%) of patients undergoing CTPA for suspected acute PE present at least one finding suggestive of chronicity [16]. Second, the subtle nature of these features often leads to initial oversight of CTEPH, resulting in considerable diagnostic accuracy variability and ultimately a delay in diagnosis [9, 15]. Compounding these drawbacks, even expert radiologists show poor inter-reader agreement [16], indicating a need for improved diagnostic imaging criteria.

In our study, we found that central acute thrombi are characterized by higher attenuation than central chronic thrombi in VNC reconstructions using dlDECT. Studies on the histopathologic composition of thrombi extracted from acute PE patients have demonstrated these thrombi to be typically acute and to contain high quantities of RBCs [8, 44, 45]. Further, previous true-non-contrast studies—both in vivo and ex vivo—have established a correlation between the attenuation of thrombi on true-non-contrast images and their RBC content [23, 24, 27]. The capability of dlDECT-based VNC reconstructions to give an estimate for true-non-contrast enhancement of various cardiovascular pathologies has been evidenced in numerous studies [46, 47]. Although lacking a direct validation with true-non-contrast images, our results suggest that the same holds true for pulmonary emboli as our findings align with the results from Luca et al., who demonstrated a higher attenuation of acute compared to chronic pulmonary thrombi in true-non-contrast images [31]. Of note, there was no evidence of a difference between VNC attenuation of segmental acute and chronic thrombi. This likely stems from multiple factors. First, the smaller and unbalanced sample size of this cohort (19 vs 5) may contribute to this finding. Second, technical considerations suggest that attenuation values in VNC images are particularly challenging to ascertain in small structures [48]. Consequently, our results suggest that VNC-reconstructions from dlDECT allow for an estimation of RBC content and thus age in large/central thrombi.

We observed that both thrombus attenuation in conventional and VMI_50keV_ reconstructions and ID were higher in acute than in chronic thrombi. These findings stand in contrast to the results of previous single- and dual-energy CT studies, which reported higher attenuation [29, 30] and/or higher contrast enhancement of chronic thrombi [30, 31]. However, the aforementioned studies have several drawbacks not only limiting their comparability with our study but also raising questions on the applicability of their findings in the setting of CTEPH. First, these studies are either limited by their small sample sizes and/or imprecise definition of chronic PE. Besides its hemodynamic definition CTEPH is clinically characterized by specific indicators of enduring thromboembolism after a minimum of three months of anticoagulation treatment [49, 50]. A definition that was not met by any of the previous studies. As imaging in these studies was performed at an earlier time point they rather captured an evolving than a fully organized thrombus. Given that the majority of pulmonary thrombi resolve over time, with angiogenesis playing a crucial role in normal thrombus resolution, thrombi examined during this transformational phase are likely to exhibit enhanced contrast agent uptake.

Contrarily, our findings of reduced iodine uptake in thrombi from CTEPH patients align with histopathological studies indicating that these thrombi are largely devoid of vascular structures [51]. In line with this observation, we detected reperfusion channels in only 5/33 (15.5%) of all chronic thrombi. Further, the reduced ID in CTEPH thrombi might reflect the decreasing intrathrombotic inflammation along with progressing thrombus organization [8].

Our finding of increased contrast enhancement in acute thrombi on the other hand mirrors the results from stroke research, indicating thrombi with higher RBC content are more permeable compared to those richer in fibrin [28, 52]. Considering the reduced iodine uptake and the lower VNC values, potentially reflecting the decreased permeability due to the low RBC density of CTEPH thrombi, and the requirement that all CTEPH patients underwent at least three months of anticoagulation prior to referral, our findings indirectly reinforce the existing evidence that thrombus permeability and attenuation are predictive of the response to thrombolytic therapy [26, 32, 44, 52, 53].

Notwithstanding these considerations, both VNC and IDO reconstructions demonstrated lesser diagnostic accuracy when compared to conventional and VMI_50keV_ reconstructions. In the light of our findings, this may be attributed to the fact that the latter methods integrate both the cellular composition and the contrast enhancement of the pulmonary thrombi. Notably, combining conventional and VMI_50keV_ reconstructions with the MPA diameter significantly improved diagnostic accuracy for distinguishing between thrombi from acute PE and CTEPH. Consequently, both parameters could effectively augment the current morphological parameters used to distinguish between both entities.

### Limitations

Besides its retrospective design, this study has several limitations that must be acknowledged. First, our assessment was solely based on ROI measurements, which potentially fall short to capture the heterogeneous composition of thrombi; nevertheless, this method demonstrated high inter-reader reproducibility. Second, a considerable proportion of patients in both the CTEPH (74/107, 69%) and the acute PE groups (73/150, 49%) were excluded due to small thrombus sizes, which limits the applicability of our results to everyday clinical practice. Third, both CTEPH and PE diagnoses were established in accordance with the respective guidelines. However, histopathological verification of thrombus age was not possible in either group. Given the diagnostic work-up of both entities, greater uncertainties regarding the true thrombus age and clot composition remain for the acute PE patients, which may affect our results. Therefore, future studies incorporating in vivo DECT-based thrombus characterization alongside histopathological thrombus analysis would be desirable to validate of our findings.

Last, although our findings align with evidence from stroke research and ex vivo investigations, they diverge in part from the prevailing literature on pulmonary thromboembolism [29, 30]. This divergence may be ascribed to the differential patient selection criteria used for the chronic PE cohort in our study. As this is the first study to adhere rigorously to the current definition of CTEPH [50], further validation of our results within a larger patient population is highly warranted. Additionally, the potential diagnostic value of our findings in identifying patients with acute on chronic PE, remains unclear based on our data and merits further investigation.

## Conclusion

In summary, thrombi in patients with CTEPH are characterized by lower attenuation in conventional CT images, VNC and VMI_50keV_ reconstructions as well as a reduced contrast enhancement.

While dlDECT reconstructions do not yield a higher diagnostic accuracy to differentiate between acute PE and CTEPH than conventional images, they provide a deeper insight into the attenuation characteristics of pulmonary emboli. Especially attenuation properties in central pulmonary thrombi can complement current morphological criteria, thereby refining diagnostic accuracy and potentially guiding therapy decisions in the context of pulmonary embolism.

## Supplementary Information

Below is the link to the electronic supplementary material.

Supplementary file1 (DOCX 237 KB)

## Data Availability

No datasets were generated or analysed during the current study.

## Abbreviations

AUC: Area under the curve
CTEPH: Chronic thromboembolic pulmonary hypertension
CTPA: CT pulmonary angiography
DECT: Dual-energy CT
dlDECT: Dual-layer dual-energy CT
ID: Iodine density
IDO: Iodine density overlay
MPA: Main pulmonary artery
PE: Pulmonary embolism
PH: Pulmonary hypertension
RBC: Red blood cell
ROI: Region of interest
RV: Right ventricle/ventricular
VMI: Virtual monoenergetic images
VNC: Virtual non-contrast
