# Supplementary material for: Dual-layer dual-energy CT characterization of thrombus composition in acute pulmonary embolism and chronic thromboembolic pulmonary hypertension
Source: Int J Cardiovasc Imaging. 2024 Dec 25;41(2):303–14. doi: 10.1007/s10554-024-03309-2 (PMC11811430; doi:10.1007/s10554-024-03309-2)
Supplement: Supplementary file 1 — Supplementary file1 (DOCX 237 KB) [file 10554_2024_3309_MOESM1_ESM.docx]

**Table S1** Intra-reader and inter-reader reproducibility.

|  | **Bland-Altman analysis** |  | **Intraclass correlation** |  |  |  |
| --- | --- | --- | --- | --- | --- | --- |
|  | **Bias (95% CI)** | **Constant bias (R^2^, p)** | **Reject ICC(1): F (p)** | **ICC(1) (95% CI)** | **ICC(C,1) (95% CI)** | **ICC(A,1) (95% CI)** |
| **Intra-reader reproducibility** |  |  |  |  |  |  |
| Conventional (n=20) | 2 HU (-5 - 13 HU) | yes (R^2^=0.02, p=0.5) | no: 2.91 (p=0.10) | 0.95 (0.89-0.98) | - | - |
| VMI_50keV_ (n=20) | 1.69 HU (-17.86 - 20.98 HU) | yes (R^2^=0.01, p=0.7) | no: 1.00 (p=0.3) | 0.94 (0.87-0.98) | - | - |
| IDO (n=20) | 0 mg/ml (0 - 0 mg/ml) | yes (R^2^=0.13, p=0.12) | no: 1.00 (p=0.3) | 0.94 (0.85-0.97) | - | - |
| VNC (n=20) | 2 HU (-7 - 12 HU) | yes (R^2^=0.00, p=1.0) | no: 2.65 (p=0.12) | 0.94 (0.85-0.97) | - | - |
| Z-Effective (n=20) | -0.01 (-0.26 - 0.29) | yes (R^2^=0.05, p=0.3) | no: 1.00 (p=0.3) | 0.92 (0.81-0.97) | - | - |
| Electron Density (n=16) | 0.11 (-1.25 - 1.17) | yes (R^2^=0.00, p=0.9) | no: 1.00 (p=0.3) | 0.91 (0.77-0.97) | - | - |
| ROI size (n=20) | 12 mm^2^ (-22 - 47 mm^2^) | no (R^2^=0.26, p=0.02) | yes: 7.36 (p=0.014) | 0.79 (0.55-0.91) | 0.84 (0.63-0.93) | 0.79 (0.47-0.92) |
| **Inter-reader reproducibility** |  |  |  |  |  |  |
| Conventional (n=20) | 2 HU (-11 - 27 HU) | yes (R^2^=0.00, p=1.0) | no: 1.00 (p=0.3) | 0.80 (0.57-0.91) | - | - |
| VMI_50keV_ (n=20) | 7.65 HU (-11.39 - 43.61 HU) | yes (R^2^=0.00, p=0.8) | yes: 5.31 (p=0.03) | 0.86 (0.68-0.94) | 0.88 (0.72-0.95) | 0.86 (0.64-0.94) |
| IDO (n=20) | 0 mg/ml (0 - 1 mg/ml) | yes (R^2^=0.10, p=0.2) | yes: 8.37 (p=0.009) | 0.81 (0.59-0.92) | 0.86 (0.68-0.94) | 0.82 (0.50-0.93) |
| VNC (n=20) | -4 HU (-25 - 5 HU) | yes (R^2^=0.00, p=0.9) | yes: 5.77 (p=0.03) | 0.85 (0.66-0.94) | 0.88 (0.71-0.95) | 0.85 (0.62-0.94) |
| Z-Effective (n=20) | 0.14 (-0.10 - 0.59) | yes (R^2^=0.01, p=0.7) | yes: 9.55 (p=0.006) | 0.80 (0.57-0.92) | 0.86 (0.67-0.94) | 0.81 (0.45-0.93) |
| Electron Density (n=16) | -0.52 (-2.43 - 0.38) | yes (R^2^=0.00, p=1.0) | yes: 5.95 (p=0.03) | 0.81 (0.55-0.93) | 0.85 (0.63-0.95) | 0.82 (0.49-0.94) |
| ROI size (n=20) | -1 mm^2^ (-18 - 5 mm^2^) | yes (R^2^=0.06, p=0.3) | no: 1.00 (p=0.3) | 0.98 (0.96-0.99) | - | - |

ICC, Intraclass correlation coefficient; HU, Hounsfield units; VMI, virtual monoenergetic images; IDO, iodine density overlay; VNC, virtual non-contrast.
